# MDM2 Antagonists Induce a Paradoxical Activation of Erk1/2 through a P53-Dependent Mechanism in Dedifferentiated Liposarcomas: Implications for Combinatorial Strategies

**DOI:** 10.3390/cancers12082253

**Published:** 2020-08-12

**Authors:** Shomereeta Roy, Audrey Laroche-Clary, Stephanie Verbeke, Marie-Alix Derieppe, Antoine Italiano

**Affiliations:** 1 Sarcoma Unit, Institut Bergonié, 33000 Bordeaux, France; shomereeta@gmail.com (S.R.); a.laroche-clary@bordeaux.unicancer.fr (A.L.-C.); s.verbeke@bordeaux.unicancer.fr (S.V.); 2 University of Bordeaux, 33400 Talence, France; 3 Sarcoma Unit, INSERM U1218, Institut Bergonié, 33000 Bordeaux, France; 4 Animalerie Mutualisée, University of Bordeaux, 33400 Talence, France; marie-alix.derieppe@u-bordeaux.fr; 5 Department of Medical Oncology, Institut Bergonié, 33000 Bordeaux, France

**Keywords:** liposarcoma, MDM2, MAPK pathway, targeted therapeutics

## Abstract

The *MDM2* gene is amplified in dedifferentiated liposarcoma (DDLPS). Treatment with MDM2 antagonists is a promising strategy to treat DDLPS; however, drug resistance is a major limitation when these drugs are used as a single agent. This study examined the impact of MDM2 antagonists on the mitogen-activated protein kinase (MAPK) pathway in DDLPS and investigated the potential synergistic activity of a MAPK kinase (MEK) inhibitor in combination with MDM2 antagonists. We identified a synergistic effect and identified the mechanism behind it. Combination effects of MDM2 antagonists and a MEK inhibitor were analyzed in a patient-derived xenograft mouse model and in DDLPS and leiomyosarcoma cell lines using different cell proliferation assays and immunoblot analysis. MDM2 antagonist (RG7388)-resistant IB115 [P4] cells and p53-silenced DDLPS cells were also established to understand the importance of functional p53. We found that MDM2 antagonists induced an upregulation of phosphorylated extracellular signal-regulated kinase (p-ERK) in DDLPS cells. The upregulation of p-ERK occurred due to mitochondrial translocation of p53, which resulted in increased production of reactive oxygen species, causing the activation of receptor tyrosine kinases (RTKs). Activated RTKs led to the activation of the downstream MEK/ERK signaling pathway. Treatment with a MEK inhibitor resulted in decreased expression of p-ERK, causing significant anti-tumor synergy when combined with MDM2 antagonists. Our results provide a framework for designing clinical studies of combination therapies in DDLPS patients.

## 1. Introduction

Dedifferentiated liposarcoma (DDLPS) is one of the most common subtypes of sarcoma. Surgical resection in reference centers is the cornerstone of DDLPS treatment [1]. However, locoregional recurrence occurs in more than 50% of cases, particularly in tumors located in the retroperitoneum. Metastatic disease is observed in up to 30% of patients [2]. Anthracycline-based chemotherapy is the standard first-line treatment in the advanced setting and is associated with very modest efficacy: the median progression-free survival for advanced DDLPS is less than 5 months [3].

*MDM2* gene (12q13-15) amplification is the genetic hallmark of DDLPS [1,4,5]. This gene encodes an E3 ubiquitin ligase that ubiquitinates and causes the degradation of the tumor suppressor protein p53 [6]. In addition, p53 binds to the promoter region of *MDM2* and enhances its transcription, thus creating an autoregulatory feedback loop that maintains the balance of p53 and MDM2 under normal physiological conditions [7]. Activation of p53 exerts significant anticancer effects, inducing apoptosis, controlling cell cycle progression, and promoting DNA repair and senescence [8]. In cells with high expression of MDM2, p53 becomes inactivated, resulting in insufficient apoptosis and cell cycle arrest [9]. Restoring the anticancer effects of p53 by blocking the MDM2–p53 interaction can be an efficient targeted approach to treat DDLPS. 

The cis-imidazolines (nutlin) compounds comprise the first class of anti-cancer agents specifically designed to inhibit the interaction between MDM2 and p53, thereby stabilizing p53 and restoring its anticancer effects [10,11]. These MDM2 antagonists bind to the p53-binding pocket of MDM2, thereby blocking the MDM2–p53 interaction and increasing p53 levels [12]. More recently, additional compounds have entered clinical development. For example, HDM201 is an imidazopyrrolidinone scaffold-based inhibitor of the MDM2–p53 interaction with high in vitro activity/selectivity and improved oral bioavailability and in vivo pharmacokinetic and pharmacodynamic profiles [13,14]. We and others have shown that only a minority of DDLPS patients treated with p53–MDM2 antagonists experienced tumor shrinkage and that the median time to disease progression was only 6 months after treatment onset. This points towards the existence of mechanisms that counterbalance the restoration of the p53 pathway [12]. 

The mitogen-activated protein kinase/extracellular signal-regulated kinase (MAPK/ERK) pathway, which is generally activated by growth factors, is known to play a crucial role in cellular proliferation, differentiation, migration, and death [15]. It has previously been reported in human blood cancer, colon cancer, and osteosarcoma that p53 stimulated the MAPK/ERK pathway, protecting the cancerous cells from p53-dependent apoptosis [15,16]. Therefore, we decided to address the impact of MDM2 antagonists on the MAPK pathway in DDLPS and to determine the molecular mechanisms underlying the potential synergy between these agents and a MAPK kinase (MEK) inhibitor. 

## 2. Results

### 2.1. A MDM2 Antagonist Induces Significant Upregulation of Phosphorylated Erk in P53 Wild-Type, But Not P53-Null, Sarcoma Cell Lines

**Figure 1:**
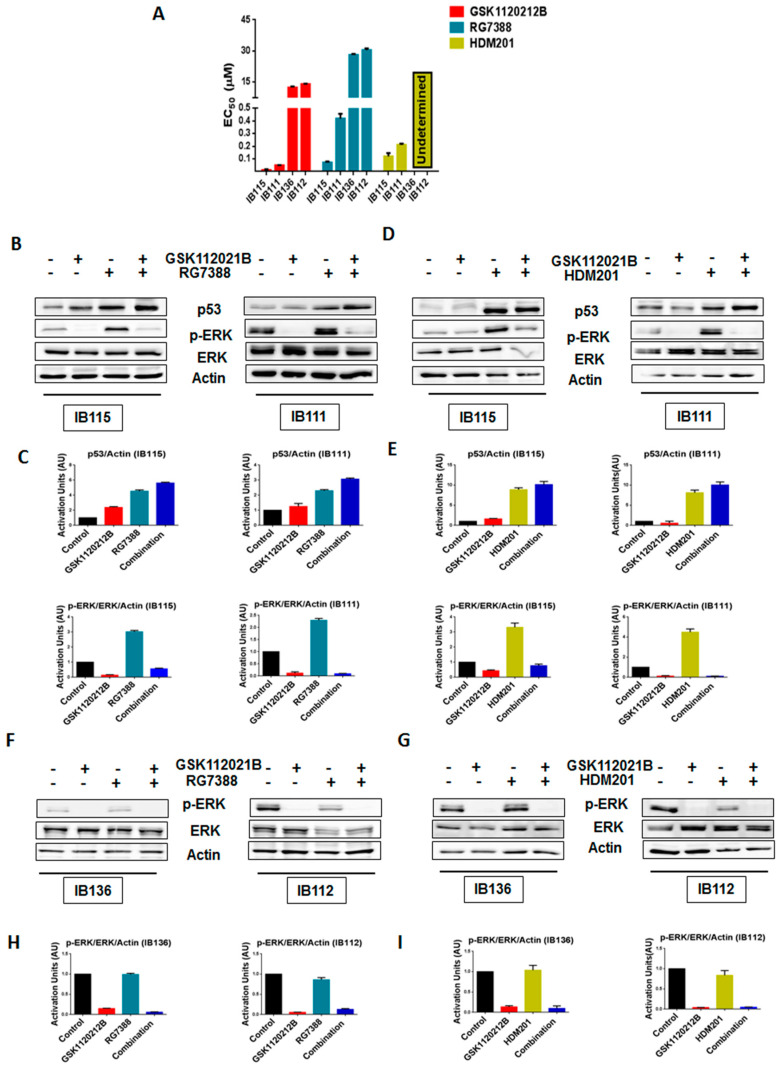
Significant upregulation of p-ERK by MDM2 antagonists is observed in p53 wild-type cells. (**A**) EC_50_ values for GSK112021B, RG7388, and HDM201 in p53 wild-type cells (IB115 and IB111) and p53-null cells (IB136 and IB112). (**B**) Representative blots of p53 wild-type cells (IB115 and IB111) treated with GSK112021B and RG7388, alone or in combination, at their EC_50_ values and immunoblotted for p53, p-ERK, and ERK. (**C**) Quantification of blots of IB115 and IB111 treated with GSK112021B and RG7388, alone or in combination. (**D**) Representative blots of p53 wild-type cells (IB115 and IB111) treated with GSK112021B and HDM201, alone or in combination, at their EC_50_ values and immunoblotted for p53, p-ERK, and ERK. (**E**) Quantification of blots of IB115 and IB111 treated with GSK112021B and HDM201, alone or in combination. (**F**) Representative blots of p53-null cells (IB136 and IB112) treated with GSK112021B and RG7388, alone or in combination, at their EC_50_ values and immunoblotted for p-ERK and ERK. (**G**) Quantification of blots of IB136 and IB112 cells treated with GSK112021B and RG7388, alone or in combination. (**H**) Representative blots of p53-null cells (IB136 and IB112) treated with GSK112021B and HDM201, alone or in combination, at their EC_50_ values and immunoblotted for p-ERK and ERK. (**I**) Quantification of blots of IB136 and IB112 cells treated with GSK112021B and HDM201, alone or in combination. Immunoblot data shown represent the results of two independent experiments.

As expected, cell viability assays revealed that IB111 and IB115 cells were exquisitely sensitive to MDM2 antagonists (RG7388 and HDM201), whereas p53-null (IB136 and IB112) cells were resistant, with undetermined HDM201 EC_50_ values for IB136 and IB112 (Figure 1A and Table S1). We performed immunoblot analysis to assess the levels of phosphorylated ERK (p-ERK) in p53 wild-type IB115 and IB111 cells and p53-null IB136 and IB112 treated with RG7388 and HDM201. We observed that exposure of IB115 and IB111 cells to RG7388 or HDM201 induced a significant increase in the expression of p-ERK, along with increase in the expression of p53 (Figure 1B–E). This increased expression of p-ERK was inhibited by treatment with GSK112021B, a selective small-molecule and adenosine triphosphate-noncompetitive inhibitor of the activation and kinase activity of MEK1 and MEK2. In contrast, we did not observe any change in the expression of p-ERK in the p53-null IB136 and IB112 cells treated with RG7388 or HDM201 (Figure 1F–I). Collectively, these results suggested that MDM2 antagonists induced a paradoxical activation of MEK/ERK signaling in DDLPS that could be mediated by p53.

### 2.2. MEK Inhibitor GSK1120212B and MDM2 Antagonists Synergistically Induce Apoptosis and G2/M Arrest Cell Cycle Arrest in p53 Wild-Type Cells 

**Figure 2:**
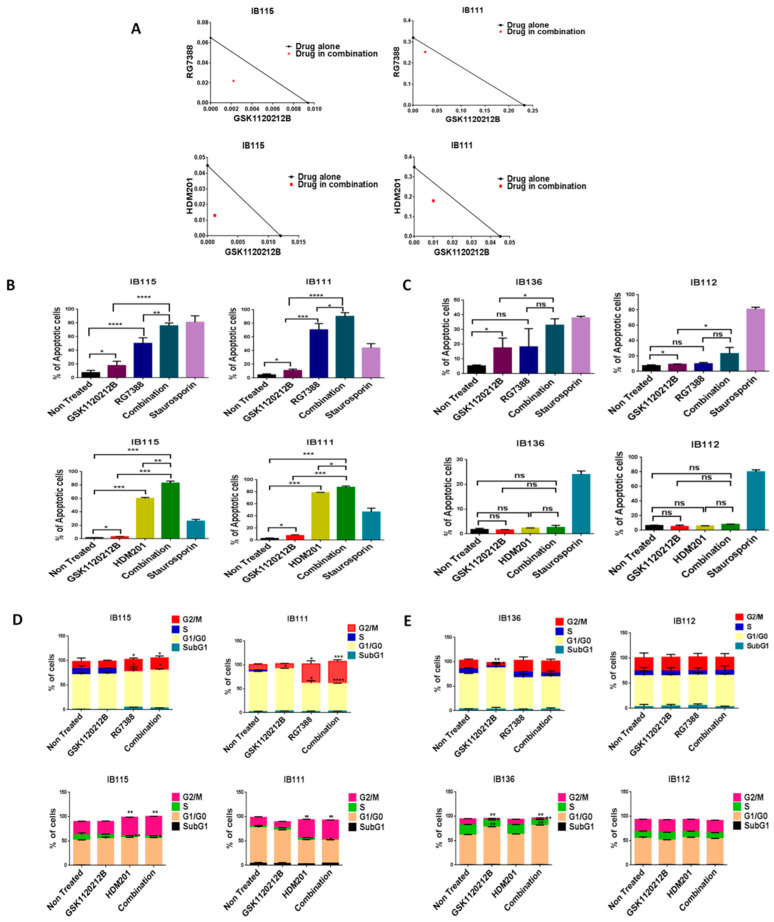
MDM2 antagonists synergize with a MEK inhibitor in p53 wild-type cells. (**A**) Isobolograms showing the synergistic effect of RG7388 and HDM201 with GSK112021B in p53 wild-type cells (IB115 and IB111). Effect of GSK112021B, RG7388, and HDM201, alone or in combination, on apoptosis in p53 wild-type cells (IB115 and IB111) (**B**) and p53-null cells (IB136 and IB112) (**C**) Effect of GSK112021B, RG7388, and HDM201, alone or in combination, on cell cycle in p53 wild-type cells (IB115 and IB111) (**D**) and p53-null cells (IB136 and IB112) (**E**) * *p* ≤ 0.05, ** *p* ≤ 0.01, *** *p* ≤ 0.001, **** *p* ≤ 0.0001, and ns (non-significant). Data shown represent the results of three independent experiments.

Since high levels of p-ERK can affect the anti-survival and pro-apoptotic effects of MDM2 antagonists, we investigated whether inhibition of p-ERK by GSK112021B could increase the response of DDLPS cells to MDM2 antagonists. We found that MDM2 antagonists (RG7388 or HDM201) and GSK112021B synergistically inhibited the growth and induced apoptosis of p53 wild-type IB115 and IB111 DDLPS cells (Figure 2A,B), but not p53-null IB112 and IB136 cells (Figure 2C). Moreover, cell cycle analysis revealed that this drug combination resulted in significant G2/M arrest in p53 wild-type IB115 and IB111 cells (Figure 2D), but not p53-null IB112 and IB136 cells (Figure 2E). These results showed the importance of the presence of intact p53 for the effectiveness of MDM2 antagonists in combination with the MEK inhibitor GSK112021B.

### 2.3. Intact p53 Is Necessary for the MDM2 Antagonist-Induced Activation of MEK/ERK Signaling in DDLPS

**Figure 3:**
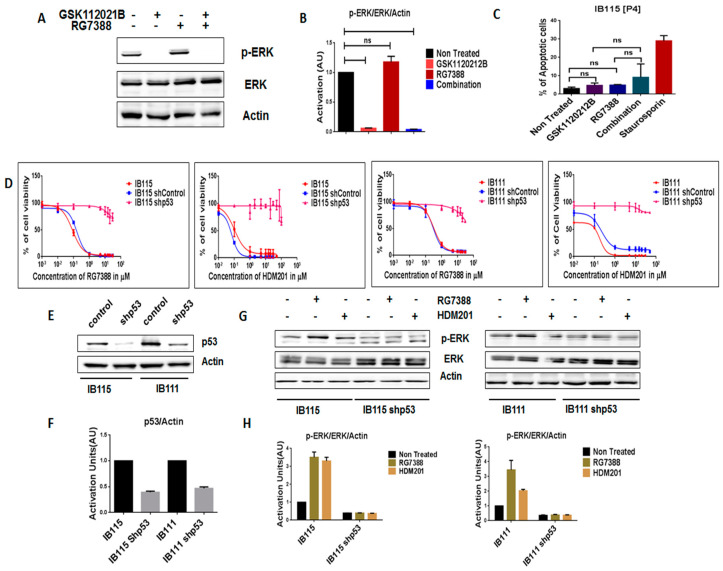
Intact functional p53 is necessary for MDM2 antagonists to activate MEK/ERK signaling in dedifferentiated liposarcoma. (**A**) Representative blots of IB115 [P4] treated with GSK112021B and RG7388, alone or in combination, at the EC_50_ values for IB115 and immunoblotted for p-ERK and ERK. (**B**) Quantification of blots of IB115 [P4] cells treated with GSK112021B and/or RG7388. (**C**) Effect of GSK112021B and RG7388, alone or in combination, at the EC_50_ values for IB115 on apoptosis in IB115 [P4] cells. (**D**) Efficacy of RG7388 and HDM201 in p53 wild-type and silenced IB115 and IB111 cells treated with increasing doses for 72 h. (**E**) Representative blots of p53 wild-type and silenced IB115 and IB111 immunoblotted for p53 to confirm p53 silencing. (**F**) Quantification of blots of p53 wild-type and silenced IB115 and IB111 cells immunoblotted for p53. (**G**) Representative blots of p53 wild-type and silenced IB115 and IB111 treated with RG7388 and HDM201 and immunoblotted for p-ERK and ERK. (**H**) Quantification of blots of p53 wild-type and silenced IB115 and IB111 cells treated with RG7388 and HDM201 and immunoblotted for p-ERK and ERK. ns (non-significant). Data represent the results of two experiments. Immunoblot data represent the results of two independent experiments and apoptosis data represent the results of three independent experiments.

In order to further assess the importance of intact p53 for the MDM2 antagonist-induced activation of MEK/ERK signaling in DDLPS, we analyzed the expression of p-ERK in RG7388-resistant IB115 [P4] cells, which were derived from IB115 cells repeatedly exposed to RG7388 and harboring a *TP53* mutation (12). As observed via immunoblot analysis, treatment of IB115 [P4] cells with RG7388 did not result in an increase in p-ERK expression (Figure 3A,B). Moreover, an apoptosis assay revealed that RG7388 and GSK112021B were not synergistic (Figure 3C) in IB115 [P4]. This is similar to the results observed in the p53-null cells (IB112 and IB136; Figure 2C).

We then investigated the impact of shRNA-mediated p53 silencing on IB115 and IB111 cells (Figure 3E,F). Using a cell viability assay, we observed that p53-silenced cells became resistant to MDM2 antagonists (Figure 3D). Moreover, they did not induce any upregulation of p-ERK, as observed in non-transfected cells (Figure 3G,H). These results confirmed our prediction that intact p53 is essential for MDM2 antagonist-induced activation of MEK/ERK signaling in DDLPS.

### 2.4. Combination Treatment of GSK1120212B and RG7388 Resulted in Decreased Tumor Volume and Increased Survival of Mice

**Figure 4:**
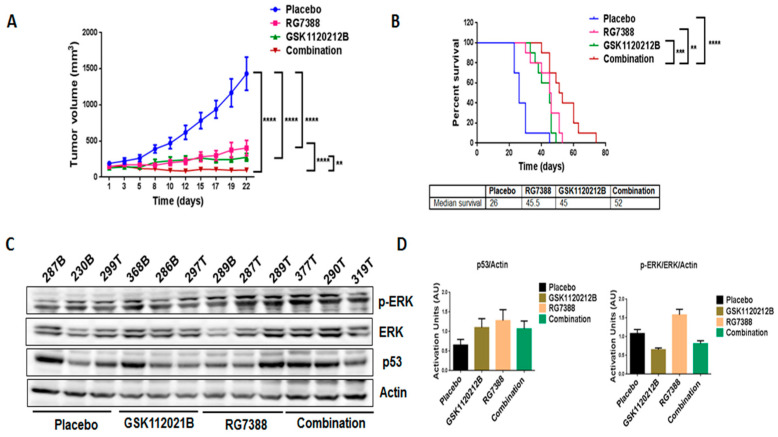
Combination treatment of GSK1120212B and RG7388 resulted in decreased tumor volume and increased survival of mice. (**A**) Effect of GSK112021B and RG7388, alone or in combination, on tumor growth. (**B**) Kaplan–Meier curve depicting overall survival of mice treated with GSK1120212B and/or RG7388. (**C**) Representative blots of tumor tissues from mice treated with GSK112021B, RG7388, or the combination and immunoblotted for p-ERK, ERK, and p53. (**D**) Quantification of blots of tumor tissues from mice treated with GSK112021B, RG7388, or the combination. ** *p* ≤ 0.01, *** *p* ≤ 0.001, and **** *p* ≤ 0.0001.

In order to validate our in vitro findings, we treated mice engrafted with IB115 cells with GSK1120212B and RG7388 as single agents and in combination. Through immunoblot analysis, it was found that, consistent with the in vitro findings, treatment with RG7388 induced a significant upregulation of MEK/ERK signaling (Figure 4C,D) that was not observed with the combination treatment. Importantly, the combination treatment significantly decreased the tumor volume (Figure 4A) and increased the survival of mice (Figure 4B) compared to treatment with single-agent GSK1120212B or RG7388. 

### 2.5. MDM2 Antagonist-Induced Activation of MEK/ERK Signaling in DDLPS Is Associated with Mitochondrial Translocation of p53 and Generation of Reactive Oxygen Species 

The results obtained led us to investigate whether MDM2 antagonist-induced activation of MEK/ERK signaling in DDLPS was related to a direct interaction between p53 and ERK or another mechanism linked the MDM2–p53 and ERK/MAPK pathways. Therefore, we performed an immunoprecipitation analysis in IB115 cells, which revealed no direct physical interaction between p53 and p-ERK, as no p-ERK bands were observed in p53-immunoprecipitated samples (Figure S1).

**Figure 5:**
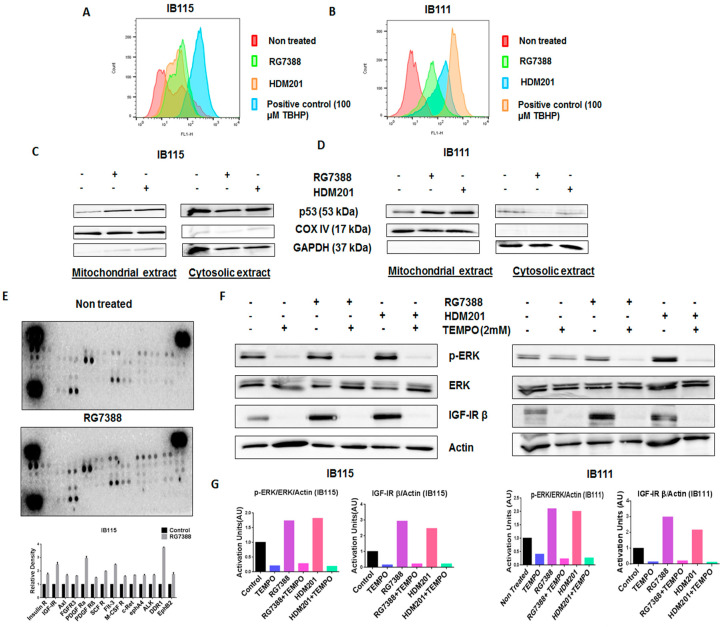
The mitochondrial translocation of p53 and p53-induced production of reactive oxygen species (ROS) upon treatment with MDM2 antagonists led to the phosphorylation of ERK in DDLPS cells through the activation of receptor tyrosine kinases (RTKs). (**A**,**B**) MDM2 antagonists RG7388 and HDM201 induced generation of ROS in IB115 and IB111 cells, which were stained with DCFDA dye for 30 min at 37 °C and treated for 4 h, and the intensities of intracellular DCF were analyzed by flow cytometer. (**C**,**D**) MDM2 antagonist (RG7388 and HDM201)-induced generation of ROS is dependent on the mitochondrial translocation of p53. IB115 (**C**) and IB111 (**D**) were treated with RG7388 and HDM201 for 24 h. After 24 h, the cells were subjected to subcellular fractionation into cytosolic and mitochondrial fractions for immunoblot analysis of p53. COX IV and GAPDH were used as mitochondrial and cytosolic loading controls, respectively. (**E**) RG7388 treatment on IB115 cells resulted in an increased expression of Insulin R and PDGFR family RTKs through Phospho-RTK array profiling. Shown is a representative example of phosphoprotein array of control and RG7388-treated IB115 using the Proteome Profiler Human Phospho-RTK Array Kit. This platform allowed simultaneous screening of 49 different RTKs. (**F**) Effect of TEMPO on RG7388- and HDM201-induced phosphorylation of ERK. DDLPS IB115 and IB111 cells were treated with 2 mM TEMPO either alone or in combination with RG7388 or HDM201 for 24 h. Cells were subjected to immunoblot analysis against p-ERK, ERK, and IGF-1Rβ. Actin was used as the loading control. (**G**) Quantification of blots of IB115 and IB111 on treatment with TEMPO, RG7388, and HDM201 alone or in combination.

Previous studies suggested that p53 promotes the production of reactive oxygen species (ROS) [17], and since ROS have been documented to activate ERK1/2 [18], we investigated the role played by ROS in RG7388- and HDM201-induced phosphorylation of ERK. The DCFDA Cellular ROS Detection Assay revealed greater accumulation of ROS in IB115 and IB111 cells treated with RG7388 and HDM201 than untreated cells (Figure 5A,B). It had been previously reported that p53 translocates to mitochondria, the main site of ROS production [19]. This led us to investigate the connection between ROS accumulation and mitochondrial translocation of p53 in IB115 and IB111 cells upon treatment with RG7388 and HDM201. Immunoblot analysis of p53 in subcellular fractions showed greater accumulation of p53 in the mitochondrial extract of IB115 and IB111 cells upon treatment with RG7388 and HDM201. COX IV was used as the loading control for the mitochondrial extract, while GAPDH was used as the loading control for cytosolic extract (Figure 5C,D). Based on the results obtained, we predicted that the mitochondrial translocation of p53 in IB115 and IB111 cells resulted in the induction of ROS and that this increased accumulation of ROS might be involved in the induction of p-ERK.

### 2.6. Phosphorylation of Receptor Tyrosine Kinases by ROS Leads to the Activation of the ERK Pathway

Our next question was how ROS were involved in the induction of p-ERK. It has been reported previously that ROS can activate receptor tyrosine kinases (RTKs) by inhibiting protein tyrosine phosphatases (PTPs). Activation of RTKs can cause phosphorylation of ERK [18]. To confirm this, we performed Human Phospho-RTK Array profiling in untreated and RG7388-treated IB115 cells. We observed upregulation of several RTKs in RG7388-treated IB115 cells, including IGF-1R, Insulin R, DDR1, and the PDGFR family of RTKs (Figure 5E).

To confirm that ROS were involved in the phosphorylation of ERK through the activation of RTKs, we used a ROS scavenger, TEMPO. DDLPS IB115 and IB111 cells were treated with 2 mM TEMPO and incubated in the presence or absence of RG7388 or HDM201 for 24 h. Immunoblot analysis revealed an upregulation of p-ERK along with the RTK IGF-1Rβ upon treatment with RG7388 or HDM201, with this upregulation of p-ERK and RTK IGF-1Rβ being significantly decreased in TEMPO-treated cells (Figure 5F,G). These findings demonstrated that MDM2 antagonist-induced ROS production and accumulation led to the phosphorylation of ERK through activation of RTKs like IGF-1Rβ.

## 3. Discussion

The *MDM2* gene has been found to be amplified in several human tumors, including in situ and invasive breast adenocarcinomas [20], esophageal cancer [21], sarcomas (either common bone and soft tissue forms) [22,23,24], and endometrial stromal tumors [25]. Importantly, MDM2 gene amplification and *TP53* mutation are usually exclusive events [24]. Therefore, several molecules have been developed to inhibit the interaction between MDM2 and p53 and to restore the antitumor activity of p53. DDLPS is one of the only tumor types (with intimal sarcomas) characterized by a consistent amplification of *MDM2* and is therefore an ideal tumor type in which to evaluate the clinical activity of such compounds. However, all of the clinical trials conducted so far reported only modest antitumor activity, with tumor shrinkage observed in only a minority of patients.

The results we report here indicate that one explanation for the limited activity of MDM2 antagonists in DDLPS is the paradoxical activation of the MAPK pathway. The MAPK pathway is known to transmit oncogenic signals, promoting cellular proliferation, differentiation, and migration and inhibiting apoptosis [15,26]. Previous studies have suggested a link between p53 signaling and the MAPK pathway. While p53 stimulated the ERK/MAPK pathway in several cancer types [15,16], Pan et al. demonstrated a p53-mediated negative regulation of the ERK/MAPK pathway using RG7388 in acute myeloid leukemia [27]. Our results showed upregulation of p-ERK when DDLPS cells were treated with two different MDM2 antagonists, RG7388 and HDM201, confirming that this event is not related to a specific chemical class of MDM2–p53 interaction inhibitors. This increase in p-ERK levels could explain the primary resistance to MDM2 antagonists observed in the majority of DDLPS patients enrolled in previous clinical studies. Limited biomarker data are available from such studies. Twenty patients with DDLPS were enrolled in a neoadjuvant biopsy-driven biomarker study investigating the impact of the MDM2 antagonist RG7112 on the p53 pathway and proliferation through sequential biopsies [10]. Interestingly, tumor biopsy samples from 6 of the 11 DDLPS patients with available pre-treatment and end of protocol biopsies exhibited an increase in Ki-67-positive cells. 

We also elucidated the possible mechanism behind the upregulation of p-ERK induced by MDM2 antagonists. We first showed that intact p53 was indispensable for the stimulation of the MAPK/ERK pathway. Since previous studies showed that upregulation of p-ERK may be related to p53-mediated production of ROS, we investigated the role of this mechanism in DDLPS. 

**Figure 6:**
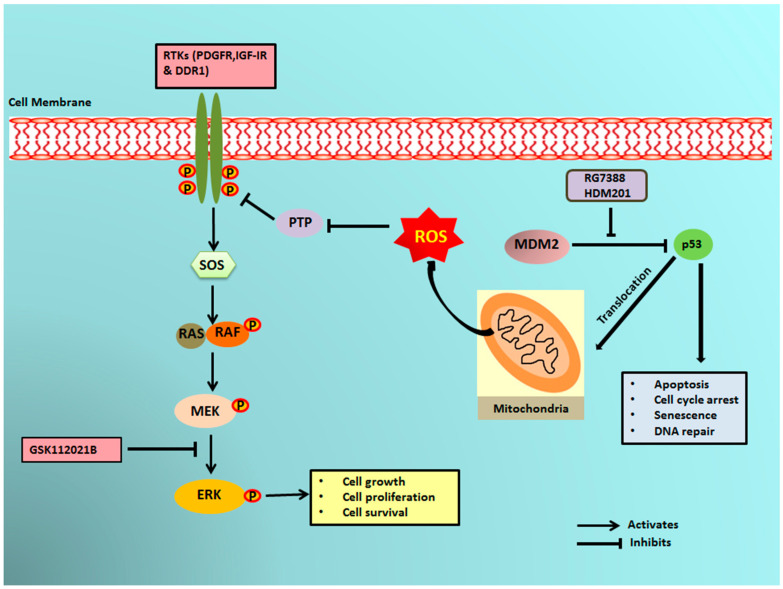
Schematic representation of MDM2 antagonist-induced phosphorylation of ERK1/2 through mitochondrial p53. MDM2 antagonists activate p53, which translocates into the mitochondria and causes elevated production of reactive oxygen species (ROS). The ROS produced inhibits the protein tyrosine phosphatases (PTPs), resulting in phosphorylation of receptor tyrosine kinases (RTKs) like PDGFR, IGF-1R, and DDR1. The activated RTKs then activate the ERK pathway.

It has previously been reported that a small portion of endogenous MDM2 can enter the mitochondria and control mitochondrial dynamics and respiration independent of p53. Mitochondrial MDM2 can enhance ROS production by repressing the transcription of the gene encoding NADH-dehydrogenase 6 (*MT-ND6*) in vitro and in vivo, affecting respiratory complex I activity [28]. Apart from MDM2, p53 can trigger the transcription of pro-oxidant genes like quinone oxidoreductase (*NQO1*, PIG3), proline oxidase (POX, PIG6), PUMA, BAX, and p66Shc and repress anti-oxidant genes like manganese superoxide dismutase (MnSOD) [17], thereby causing an imbalance between ROS production and ROS scavenging. This imbalance leads to accumulation of ROS. It has been seen that in response to any kind of stress, there is an upregulation of p53 expression, and a fraction of this elevated p53 (2%) translocates to mitochondria [17,29,30]. The p53 that translocates into the mitochondria binds to MnSOD and inhibits its ROS scavenging activity, promoting ROS generation [19]. ROS can increase phosphorylation of RTKs by inhibiting PTPs, which in turn activate Ras and, subsequently, the MAPK/ERK pathway [18,31]. Our results showed that upregulation of p53 resulting from treatment of DDLPS with MDM2 antagonists induced an upregulation of ROS through mitochondrial translocation of p53. Interestingly, we observed that treatment of DDLPS with MDM2 antagonists induced an upregulation of RTKs, including IGF-1R and PDGFRβ. These results are in line with those of previous studies showing that nutlin increased activation of RTKs such as IGF-1R in other tumor models [32]. Immunoblot analysis revealed increased expression of p-ERK along with IGF-1R β in MDM2 inhibitor-treated IB115 and IB111 cells, which was not observed when these cells were co-treated with the ROS scavenger TEMPO. Our results confirmed that MDM2 antagonists caused accumulation of p53 that translocated inside the mitochondria and caused elevated generation of ROS. This increased production of ROS resulted in phosphorylation of RTKs, perhaps through inhibition of PTPs. Activation of RTKs then led to the activation of the MAPK/ERK pathway (Figure 6).

These findings have important clinical implications. Indeed, identifying synergistic combinations is crucial to developing successful MDM2 antagonist-based therapeutic strategies for liposarcoma. Previous studies have reported that the combination of nongenotoxic nutlin with genotoxic drugs synergistically activated p53 functions, paving the way for the possible use of nutlin in combinatorial drug therapy [3,33]. The effect of combination treatment of nutlin with cytotoxic drugs currently in use (e.g., cisplatin, methotrexate, and doxorubicin) on sarcoma cells was investigated by Ohnstad et al. [10,34]. Of all the drugs tested, doxorubicin is the only drug that is used in the treatment of DDLPS. The authors observed a synergy that bolstered the development of this combination in the clinic. Our group recently reported that, in a phase 1 study, combination therapy of the nutlin compound RG7112 with doxorubicin potentiated p53 activation in a randomly selected patient population with advanced soft tissue sarcoma [35]. However, the combination therapy led to a high rate of grade 3 and 4 hematological toxicity, with 60% and 45% of patients experiencing acute neutropenia or thrombocytopenia, respectively, blocking its future clinical development. Therefore, combination therapy using nutlin and targeted nongenotoxic drugs may be a more appropriate approach. Our data provide the first in vivo evidence that inhibition of MAPK signaling can augment anti-tumor activity of a MDM2 antagonist in human tumor models.

## 4. Materials and Methods

### 4.1. Cells and Cell Culture

The DDLPS (IB115 and IB111) and leiomyosarcoma (IB136 and IB112) cell lines used in this study were derived from human surgical specimens (Institut Bergonié, Bordeaux, France) after obtaining patient consent. The MDM2 antagonist-resistant IB115 [P4] cell line was derived from IB115 cells through repeated exposure to the nutlin compound RG7388, as previously described [12]. Cells were maintained in RPMI medium 1640 (Sigma Life Technologies, St. Louis, MO, USA) with 10% fetal calf serum (Dutscher, France) in a humidified incubator containing 5% CO_2_ maintained at 37 °C.

### 4.2. Reagents

RG7388 was supplied by Roche (Roche Pharma Research & Early Development, Basel, Switzerland) and was dissolved in the solution supplied by Roche to a concentration of 10 mM as a stock solution and stored at −20 °C. GSK1120212 (MEK inhibitor) and HDM201 were purchased from Selleck Chemicals (Houston, TX, USA) and Novartis (Basel, Switzerland), respectively and were prepared as 10 mmol/L stock solutions in DMSO and stored at −20 °C. 2,2,6,6-Tetramethylpiperidine-1-oxyl (free radical; (TEMPO) was purchased from Sigma-Aldrich.

### 4.3. Cell Viability and Synergy Assay

The effects of GSK112021B, RG7388, and HDM201 on cell viability were investigated using the 3-(4, 5-dimethylthiazol-2-yl)-2, 5-diphenyl tetrazolium bromide (MTT) assay (Sigma-Aldrich Chimie, Saint-Quentin-Fallavier, France). Briefly, cells were seeded at a density of 3000 cells/well in 96-well plates and incubated for 24 h. Cells were then treated with increasing concentrations of the respective compounds for 72 h at 37 °C. After 72 h, MTT at a concentration of 0.5 mg/mL was added to each well and was incubated for 3–4 h to allow the formation of formazan crystals. The crystals were dissolved in DMSO and the absorbance of the colored solution was measured on a microplate-photometer (Bio-Tek Instruments, Colmar, France) using a test wavelength of 570 nm and a reference wavelength of 630 nm. The concentrations of substance required for 50% growth inhibition (EC_50_) were estimated using GraphPad Prism software (GraphPad Software Inc., San Diego, CA, USA). 

To measure synergistic effects between two drugs, a diagonal constant ratio combination design was used according to the protocol by Chou and Talalay [36]. Cells were incubated in a 2-fold serial dilution of the two drugs at a constant ratio, with several concentrations above and below the EC_50_ values of the drugs. After 72 h of incubation, MTT was immediately added to the wells and the absorbance was measured. The analysis of synergy was performed using the isobologram and combination index (CI) methods derived from the median effect principle of Chou and Talalay [36]. The combination effects of two compounds can be summarized as follows: CI < 1 (under the curve), CI = 1 (near the curve), and CI > 1 (above the curve) indicate synergistic, additive, and antagonistic effects, respectively.

### 4.4. Apoptosis

For apoptosis analysis, 2 × 10^5^ cells/well were seeded in a 6-well plate. After 24 h, cells were treated with the EC_50_ value of the compounds alone or in combination for 72 h at 37 °C. After 72 h, cells were exposed to FITC-Annexin V and propidium iodide (PI) according to the manufacturer’s protocol (BD Biosciences, San Jose, CA, USA). Cells were analyzed by flow cytometry using FL1 for Annexin V and FL2 for PI. The flow cytometry data (FACS Calibur; BD Biosciences) were analyzed with FlowJo v.7.6.3 software (Version 10.2. Ashland, OR: Becton, Dickinson and Company; 2019.).

### 4.5. Cell Cycle Analysis

Cells were seeded at a density of 2 × 10^5^ cells/well followed by starvation for 6 h. The cells were then treated with the EC_50_ values of the compounds alone or in combination for 48 h. After 48 h, cells were harvested and centrifuged at 1500× *g* for 5 min and washed twice with phosphate-buffered saline (PBS). Cells were then fixed and permeabilized with 70% ethanol at 4 °C overnight. Ethanol was removed and cells were washed twice with PBS. Next, 300 µL of a propidium iodide- and ribonuclease-containing solution were added to cells, which were then incubated in the dark for 30 min, followed by analysis by flow cytometry (FACS Calibur; BD Biosciences). The data obtained were analyzed with FlowJo v.7.6.3. software and results were expressed as percentage of cells in a given phase of the cell cycle.

### 4.6. Immunoblot

#### 4.6.1. Protein Extraction from Cells

Non-treated and 24 h-treated cells were harvested in radioimmunoprecipitation assay lysis buffer (150 mM NaCl, 50 mM Tris pH 7.5, 1% NP-40) containing a mix of protease inhibitors (Roche) and phosphate inhibitors (1 mM NaO and 1 mM NaF). The lysate was centrifuged at 13,000 rpm for 10 min at 4 °C and the supernatant was collected and stored at −20 °C.

#### 4.6.2. Protein Extraction from Mouse Xenograft Tumors

Frozen tumor tissues stored in liquid nitrogen were broken into small pieces, and the piece to be lysed was weighed. To the tissues, 5 µL/mg of lysis buffer (150 mM NaCl, 10 mM Tris pH 7.5, 1 mM EDTA, 1% NP-40, NaO, and 10 mM NaF containing a mix of protease inhibitors [Roche]) was added. The tissues were homogenized in the lysis buffer using a tissue homogenizer. The lysates were incubated in ice for 30 min followed by centrifugation at 13,000 rpm for 15 min at 4 °C. The supernatant was collected and stored at −20 °C.

#### 4.6.3. Mitochondrial/Cytosolic Fraction Isolation and Immunoblot Analysis

The mitochondrial/cytosolic fractions from IB115 and IB111 cells were isolated according to the manufacturer’s protocol (Mitochondria/Cytosol Fractionation Kit, ab65320 (Abcam, Cambridge, MA, USA). Next, 30 µg of protein extract was separated on SDS-polyacrylamide gel and transferred onto PVDF membrane. The membrane was blocked with 5% blocking solution for 1 h at room temperature (RT) and then probed with the primary antibody (1:1000) at 4 °C overnight. The membrane was washed with PBS with 0.1% Tween 20 and probed with horseradish peroxidase (HRP)-conjugated secondary antibody (1:5000) for 2 h at RT. The blots were visualized on a Fusion FX7 imaging system (Fisher Bioblock Scientific, Waltham, MA, USA) using the Immobilon^TM^ Western enhanced chemiluminescence detection kit (Millipore Corporation, Billerica, MA, USA). The bands obtained were analyzed and quantified using ImageJ^®^ 1.49g software (National Institutes of Health, Bethesda, MD, USA). The primary antibodies used were: anti-p53 (1:200, Santa Cruz sc-126); anti-p-ERK1/2 thr202/tyr204 (1:1000, CST 4370); anti-ERK1/2 (1:1000 ab17942); anti-IGF-1R (1:1000 CST 3027), anti-Actin (1:1000 Sigma Aldrich A3853); anti-COX IV (1:1000, CST 4844) and anti-GAPDH (1:1000, Santa Cruz sc-51907). HRP-conjugated secondary antibodies against rabbit or mouse IgG were obtained from Santa Cruz. Glycine buffer (0.15 M, pH 2) was used to strip the membranes.

### 4.7. Cellular ROS Detection Assay

For this assay, 1.5 × 10^4^ cells/well were seeded in a 6-well plate. After 24 h, cells were trypsinized and harvested. The harvested cells were stained according to the manufacturer’s protocol (DCFDA Cellular ROS Detection Assay Kit, ab113851). Cells stained with 100 µM tert-butyl hydrogen peroxide (TBHP) for 4 h before staining with DCFDA dye were the positive control.

### 4.8. Human Phospho-RTK Array 

Non-treated and treated cells were solubilized in the lysis buffer provided in the kit (Human Phospho-RTK Array Kit, R&D Systems, catalog number ARY001B). The lysates were centrifuged at 14,000× *g* for 5 min at 4 °C and the supernatant was collected and stored at −80 °C. The assay was performed according to the manufacturer’s protocol.

### 4.9. Gene Silencing by Lentiviral Infection

The pLVTH-sip53 vector expressing green fluorescent protein (GFP) and a short hairpin RNA targeting *TP53* (plasmid 12239) and control vector pLVTH (plasmid 12262) were obtained from Addgene. Viral particles were produced by calcium phosphate transfection of 293T cells. Infections were performed at a multiplicity of 10 infectious units per cell. The efficacy of infection was checked by assessing GFP expression via flow cytometry and p53 silencing was assessed by immunoblot.

### 4.10. Immunoprecipitation

Cell lysates were prepared using RIPA lysis buffer. The lysates were precleared at 4 °C for 30 min by adding 1 µg of IgG2a together with 20 µL of re-suspended volume of Protein A/G PLUS-Agarose. The beads were pelleted at 2500 rpm for 5 min at 4 °C and the precleared supernatant was collected. Next, 1 µg of primary antibody was added to 500 µg of cellular protein and was incubated for 1 h at 4 °C. Next, 20 µL of re-suspended volume of Protein A/G PLUS-Agarose was added and incubated at 4 °C on a rocker platform overnight. The immunoprecipitants were collected by centrifugation at 2500 rpm for 5 min at 4 °C. The supernatant was discarded carefully and the pellet obtained was washed with PBS. After the final wash with PBS, the pellet was resuspended in electrophoresis buffer and immunoblot was performed.

### 4.11. Animal Studies

All animal experiments were performed with the approval of the institutional animal use and care committee under project license APAFIS#8415-2017010211442345 (University of Bordeaux). This study followed the French and European Union guidelines for animal experimentation (RD 1201/05, RD 53/2013 and 86/609/CEE, respectively). IB115 cells (5 × 10^6^ cells/200 µL) were inoculated subcutaneously into the right flank of RagΥ2C −/− mice (*n* = 10 per group). Once palpable, tumor volumes were calculated using the following formula: length × width^2^/2. Once the average size of the tumors was 100 mm^3^, animals were treated with RG7388 and GSK112021B via oral gavage. The mice were randomized into four groups: vehicle, RG7388 alone (25 mg/kg, oral gavage five times per week), GSK112021B alone (0.5 mg/kg oral gavage five times per week), and both drugs (RG7388 and GSK112021B five times per week at 25 and 0.5 mg/kg, respectively). RG7388 was dissolved in a solution supplied by Roche. GSK1120212 was prepared by dissolving in 1 volume of 1-methyl-2-pyrrolidone (NMP) in a 100 °C water bath followed by the addition of 9 volumes of PEG300 (Sigma-Aldrich, St Quentin Fallavier, France). Mice in each group were treated for 3 weeks, after which treatment was stopped and tumors were measured every 2–3 days with calipers and the diameters were recorded. Mice were euthanized when the tumor volume reached 2000 mm^3^. Tumor progression was analyzed with GraphPad Prism software, and Kaplan–Meier curve analysis was used to compare the overall survival. Log-rank (Mantel–Cox) tests were used to compare Kaplan–Meier curves, and *p*-values of 0.05 and below were considered statistically significant.

### 4.12. Statistical Analysis

GraphPad Prism software was used for statistical analysis. Two-way analysis of variance and Student’s *t*-test was performed for more than two groups. All of the experiments were repeated in duplicate or triplicate. Data are represented as mean ± standard deviation and significant differences are indicated as * *p* ≤ 0.05, ** *p* ≤ 0.01, *** *p* ≤ 0.001, and **** *p* ≤ 0.0001, with ns indicating lack of statistical significance. The analysis of progression-free survival was performed using log-rank (Mantel–Cox) test.

## 5. Conclusions

Our data provide a rationale to assess the therapeutic potential of MEK inhibitors in enhancing the effect of antagonists of MDM2–p53 in DDLPS. Due to the dismal prognosis of this disease, confirming our data in the clinical setting would represent a strong accomplishment for the sarcoma community.

## Supplementary Materials

The following are available online at https://www.mdpi.com/2072-6694/12/8/2253/s1, Figure S1: Immunoprecipitation (IP) analysis revealed no direct physical interaction between p53 and p-ERK. Full blots of all immunoblots, Table S1: EC_50_ values for GSK112021B, RG7388, and HDM201 in p53 wild-type cells (IB115 and IB111) and p53-null cells (IB136 and IB112).
