# Supplementary material for: MDM2 Antagonists Induce a Paradoxical Activation of Erk1/2 through a P53-Dependent Mechanism in Dedifferentiated Liposarcomas: Implications for Combinatorial Strategies
Source: Cancers (Basel). 2020 Aug 12;12(8):2253. doi: 10.3390/cancers12082253 (PMC7465494; doi:10.3390/cancers12082253)
Supplement: Supplementary file 1 [file cancers-12-02253-s001.pdf]

*cancers*

*Article*

# MDM2 Antagonists Induce a Paradoxical Activation of Erk1/2 through a P53-Dependent Mechanism in Dedifferentiated Liposarcomas: Implications for Combinatorial Strategies

**Shomereeta Roy, Audrey Laroche-Clary, Stephanie Verbeke, Marie-Alix Derieppe and Antoine Italiano**

**Supplementary Material**

**Table S1.** $EC_{50}$ value of GSK112021B, RG7388 and HDM201 in p53 wildtype cells (IB115 and IB111) and p53 null cells (IB136 and IB112).

| Cell line | GSK1120212B (EC50) | RG7288 (EC50) | HDM201 (EC50) |
|---|---|---|---|
| IB115 (p53 wildtype) | 0.01 µM | 0.08 µM | 0.1 µM |
| IB111 (p53 wildtype) | 0.04 µM | 0.43 µM | 0.2 µM |
| IB136 (p53 null) | 12.87 µM | 28.17 µM | Undetermined |
| IB112 (p53 null) | 14.22 µM | 30.65 µM | Undetermined |

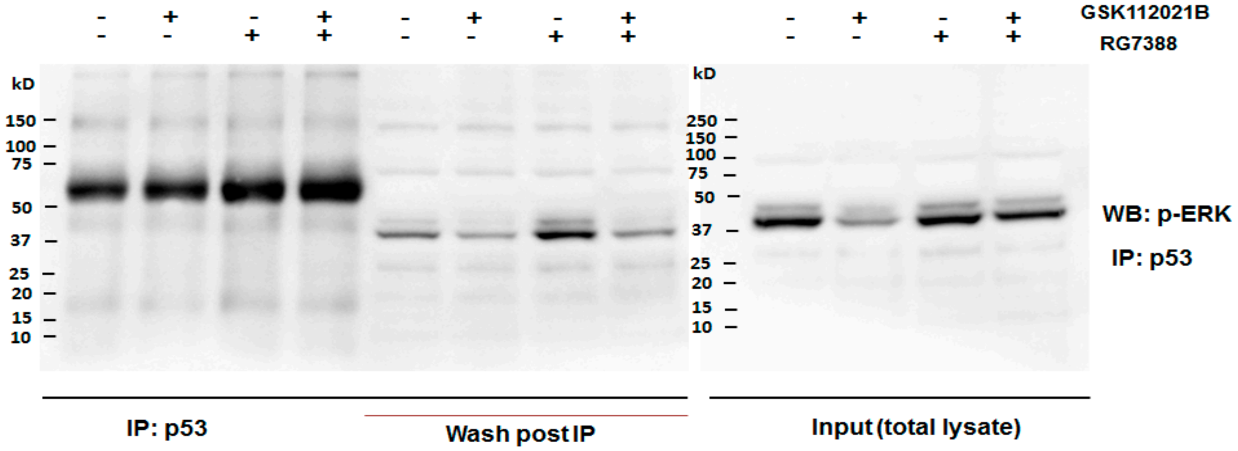


**Figure S1.** Immunoprecipitation (IP) analysis revealed no direct physical interaction between p53 and p-ERK. p53 was immunoprecipitated from protein extract of IB115 cells with different treatments. The IP lysates of different treatments immunoprecipitated with p53 showed no bands of p-ERK on immunoblotting with p-ERK. Bands of p-ERK were obtained in the 1st wash of all lysates post p53 immunoprecipitation confirming no physical interaction between p53 and p-ERK. Total protein lysates of different treatments served as the input controls.

## Methods

### *Immunoprecipitation*

Cell lysates were prepared using RIPA lysis buffer. The lysates were precleared at 4°C for 30 minutes by adding 1 µg of IgG2a together with 20µl of re-suspended volume of Protein A/G PLUS-Agarose. The beads were pelleted at 2500 rpm for 5 mins at 4°C and the precleared supernatant was collected. 1µg of primary antibody was added to 500 µg of cellular protein and was incubated for 1 h at 4°C. 20µl of re-suspended volume of Protein A/G PLUS-Agarose was added and incubated at 4°C on a rocker platform overnight. The immunoprecipitants were collected by centrifugation at 2500 rpm for 5 minutes at 4°C. The supernatant was discarded carefully and the pellet obtained was washed with PBS. After the final wash with PBS the pellet was resuspended in electrophoresis buffer and western blot was performed

## Figure 1A and 1D full blots

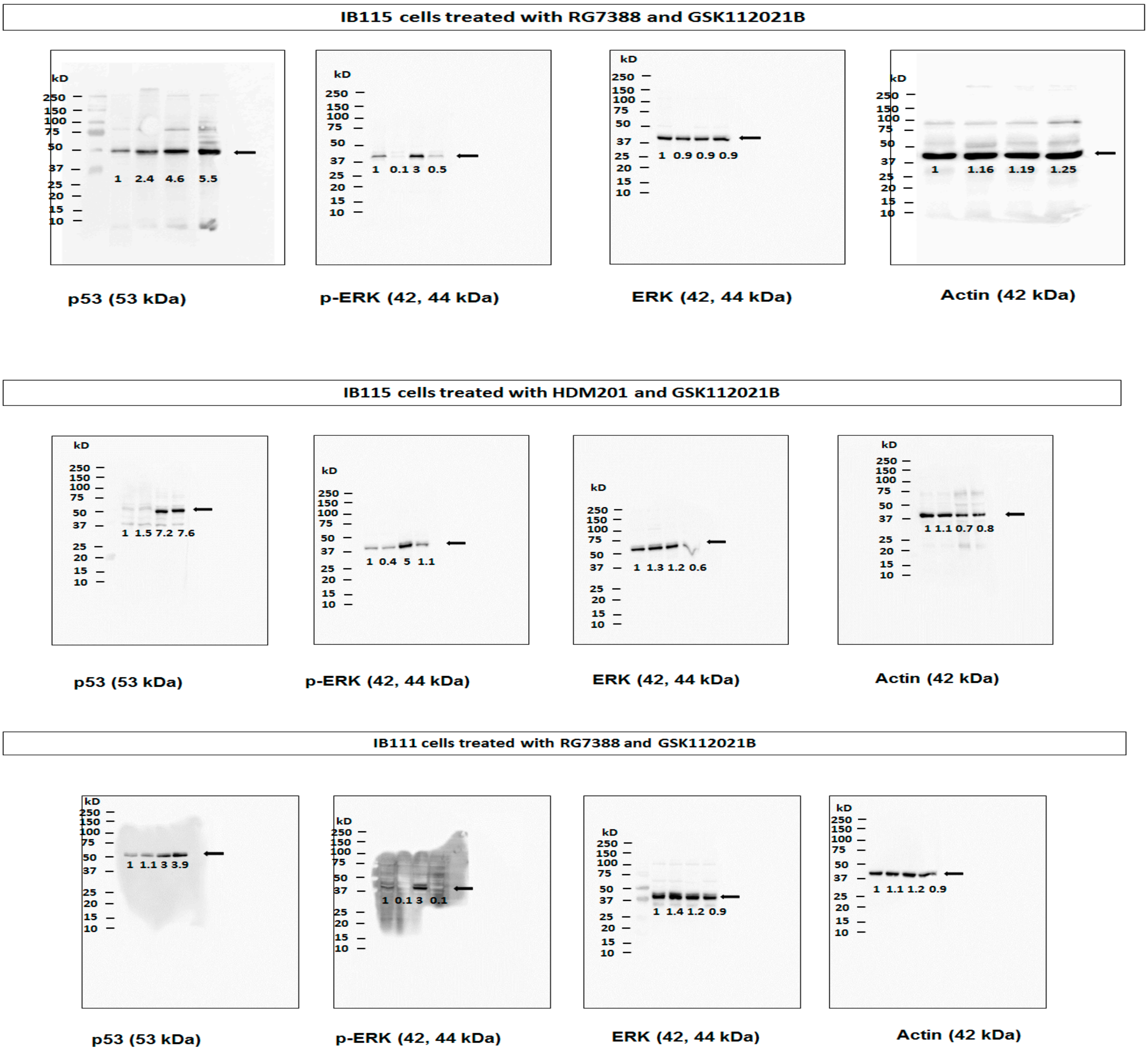

**IB111 cells treated with HDM201 and GSK112021B**

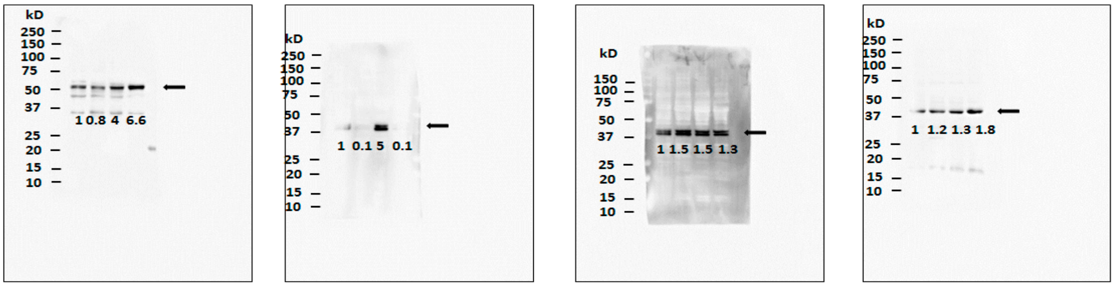


p53 (53 kDa) p-ERK (42, 44 kDa) ERK (42, 44 kDa) Actin (42 kDa)

## Figure 1F and 1G full blots

**IB136 and IB112 cells treated with RG7388 and GSK112021B**

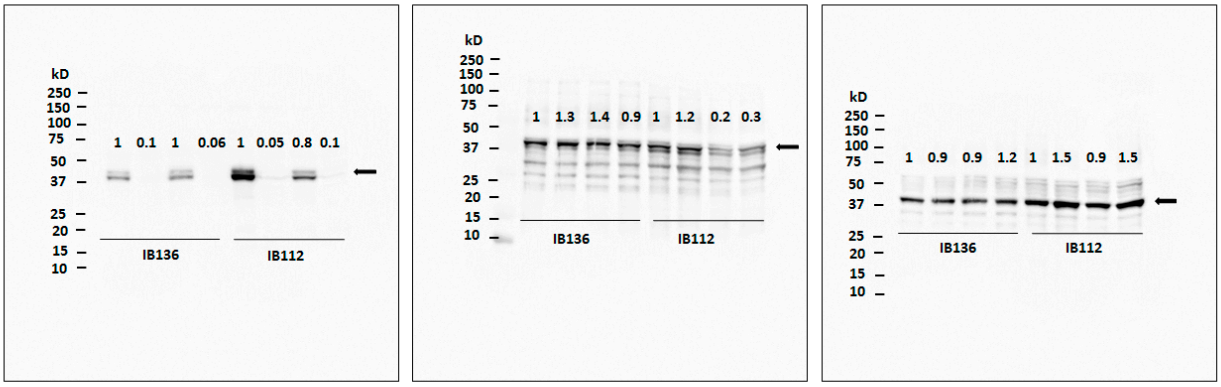


p-ERK (42, 44 kDa) ERK (42, 44 kDa) Actin (42 kDa)

**IB136 cells treated with HDM201 and GSK112021B**

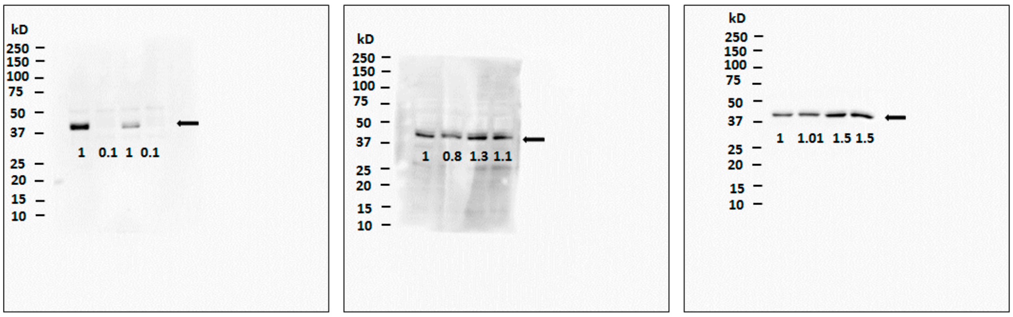


p-ERK (42, 44 kDa) ERK (42, 44 kDa) Actin (42 kDa)

**IB112 cells treated with HDM201 and GSK112021B**

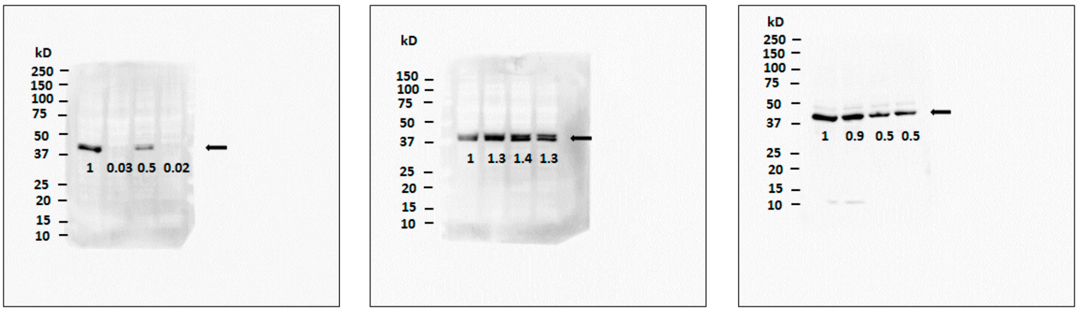


**p-ERK (42, 44 kDa)** **ERK (42, 44 kDa)** **Actin (42 kDa)**

## Figure 3A full blots

**IB115 [P4] cells treated with RG7388 and GSK112021B**

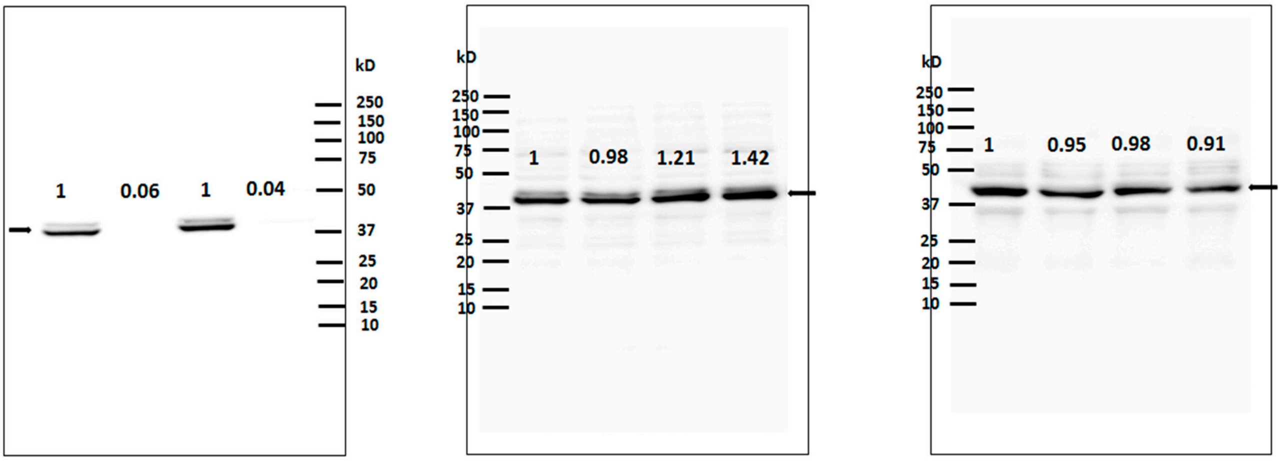


**p-ERK (42, 44 kDa)** **ERK (42, 44 kDa)** **Actin (42 kDa)**

**Figure 3E full blots**

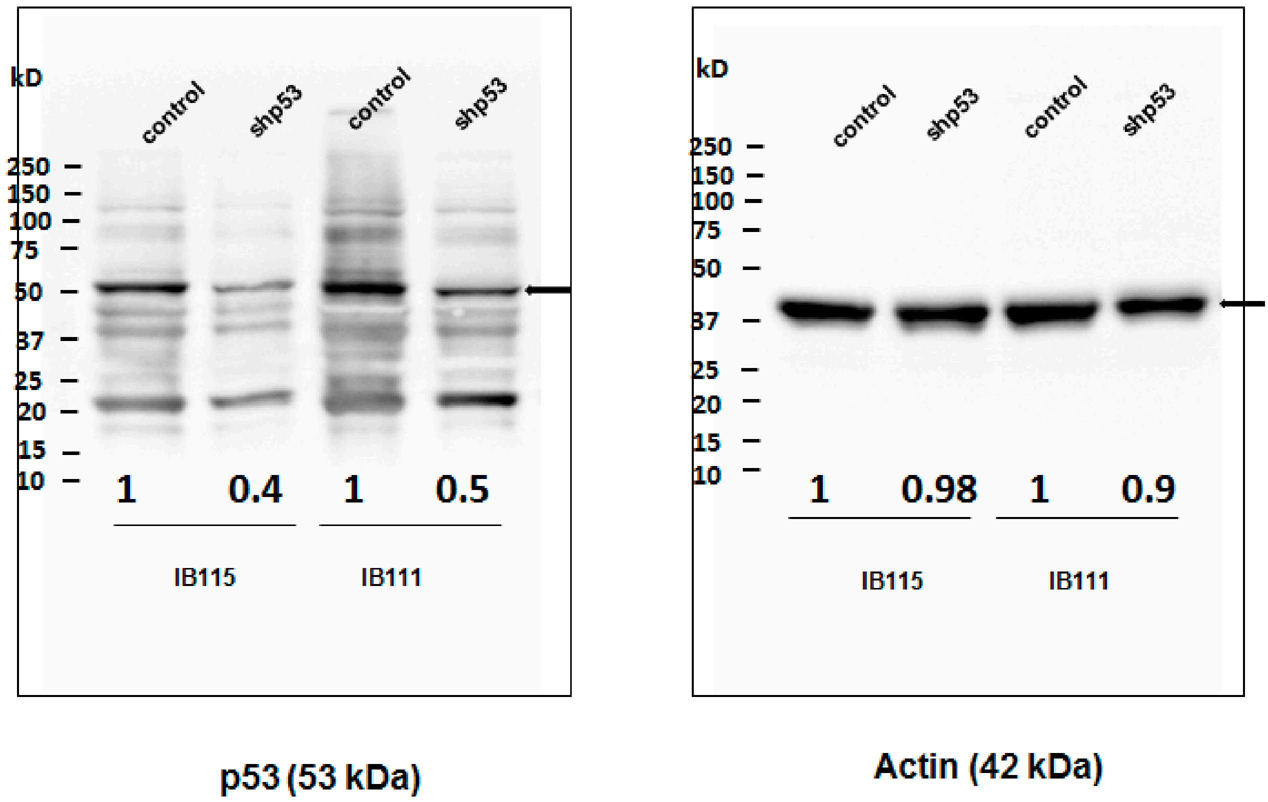


**Figure 3G full blots**

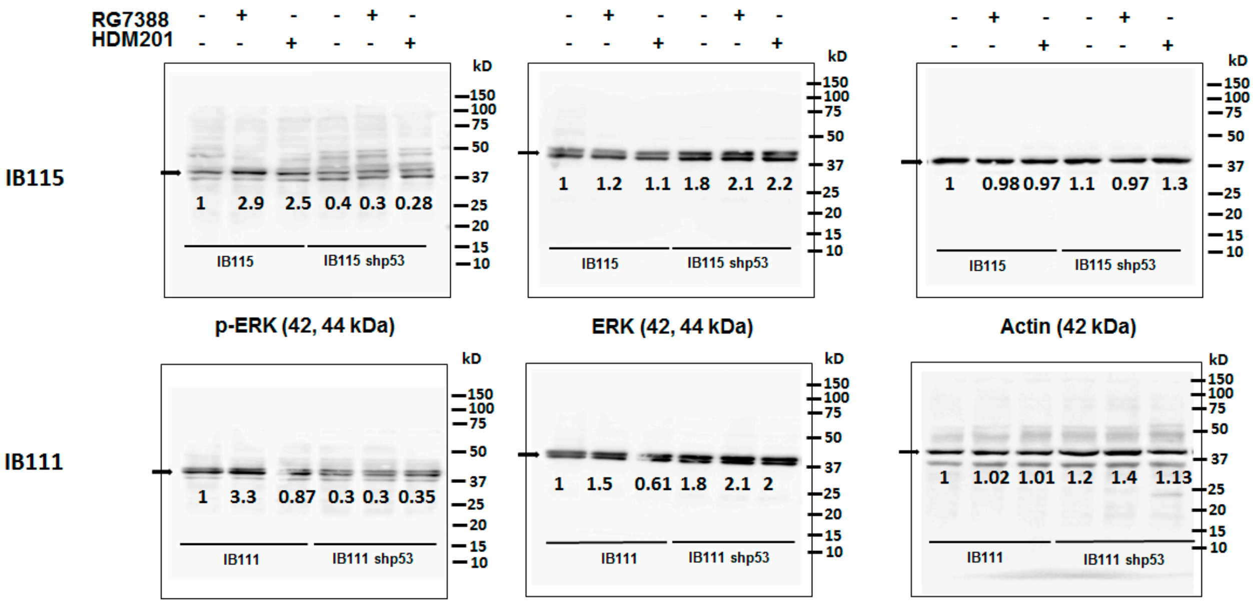

## Figure 4C full blots

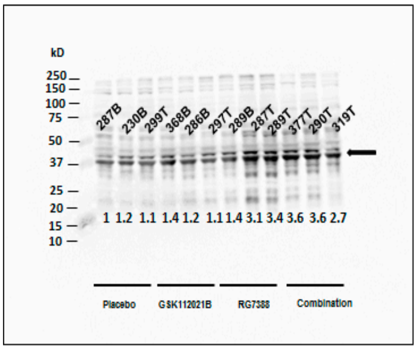


**p-ERK (42, 44 kDa)**

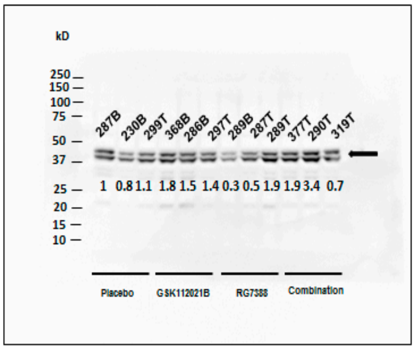


**ERK (42, 44 kDa)**

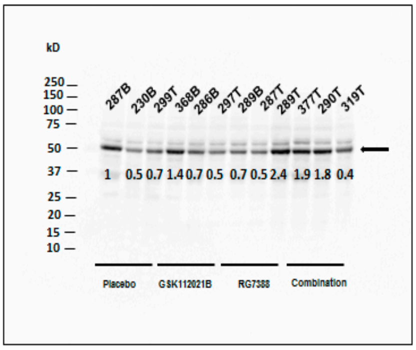


**p53 (53 kDa)**

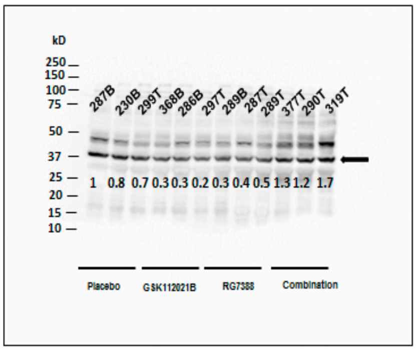


**Actin (42 kDa)**

## Figure 5C and 5D full blots

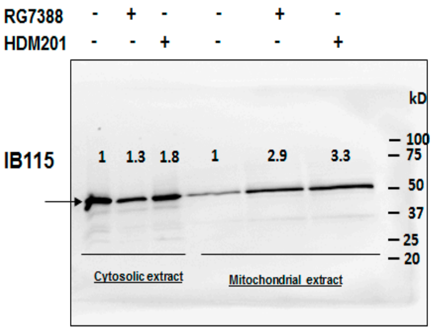


**p53 (53 kDa)**

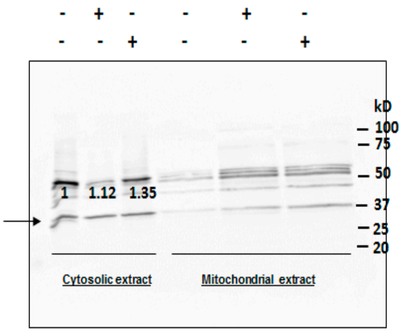


**GAPDH (37 kDa)**

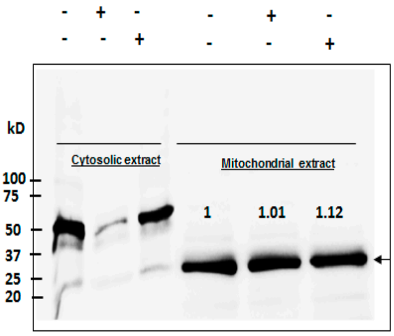


**COX IV (17 kDa)**

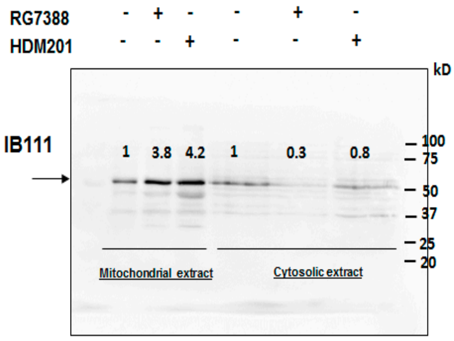


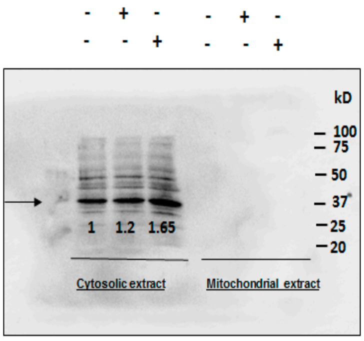


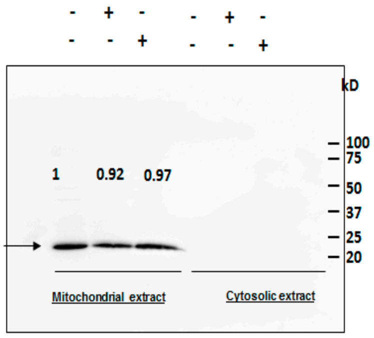

**Figure 5F full blots**

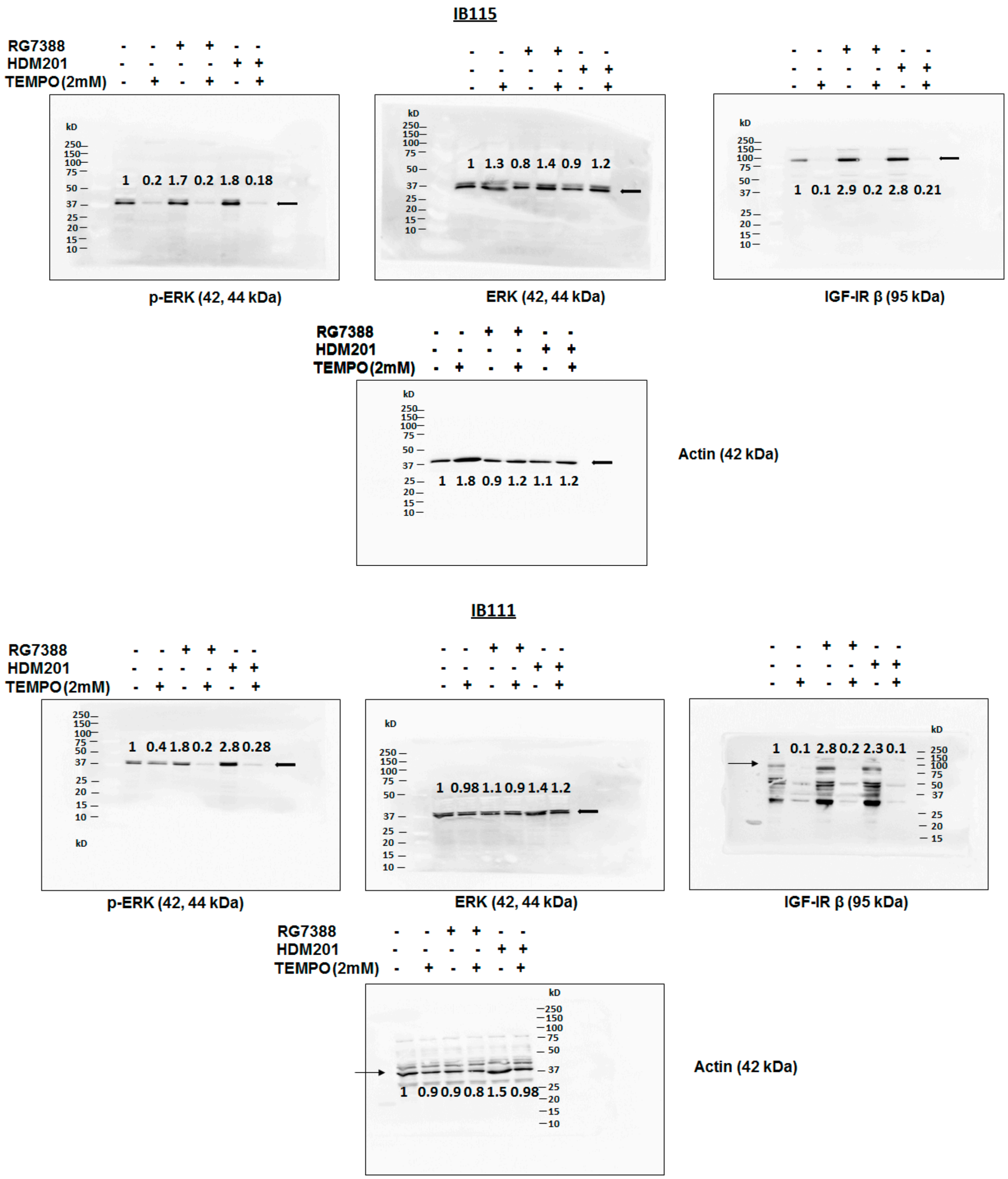


© 2020 by the authors. Licensee MDPI, Basel, Switzerland. This article is an open access article distributed under the terms and conditions of the Creative Commons Attribution (CC BY) license (http://creativecommons.org/licenses/by/4.0/).
